# Effects of Music Therapy During Total Knee Arthroplasty Under Spinal Anesthesia: A Prospective Randomized Controlled Study

**DOI:** 10.7759/cureus.7396

**Published:** 2020-03-24

**Authors:** Promil Kukreja, Katherine Talbott, Lisa MacBeth, Elie Ghanem, Adam B Sturdivant, Alexander Woods, William A Potter, Hari Kalagara

**Affiliations:** 1 Anesthesiology and Perioperative Medicine, University of Alabama at Birmingham, Birmingham, USA; 2 Orthopaedic Surgery, University of Alabama at Birmingham, Birmingham, USA

**Keywords:** music therapy, spinal anesthesia, perioperative anxiety, patient satisfaction

## Abstract

Purpose

Music therapy is an effective non-pharmacologic intervention that is cost-effective, easy to implement, and customize. It has been shown to significantly alleviate anxiety and improve patient satisfaction. In this study, we aimed to compare music therapy to a control (no music) group with respect to sedation requirements, anxiety levels, and patient satisfaction for patients undergoing total knee arthroplasty under spinal anesthesia.

Methods

In this randomized controlled study, we compared the effect of music therapy in patients ≥ 18 years old. Patients undergoing total knee arthroplasty were screened for the study to rule out any contraindications for spinal anesthesia. Patients were randomized in a 1:1 ratio for either the "music" or "control" group. Both groups were compared for sedation requirements, preoperative and postoperative anxiety levels, and patient satisfaction.

Results

Subjects in the music group had a statistically significant lower than average State-Trait Anxiety Inventory (STAI)-State baseline score as compared to the control group (music group 31.00 (standard deviation (SD) 1.44), control group 38.04 (SD 2.35); p = 0.01). Postoperative STAI-State-Trait Anxiety Inventory (STAI)-State scores for the music group were lower for the music group than the control group (music group 28.34 (SD 1.64), control group 32.21 (SD 1.56), p= 0.09). STAI-Trait scores were similar pre-operatively, but significantly less post-operatively in the music group (28.14 SD 1.0) as compared to the control group (34.71 SD 2.31); p = 0.01. Propofol dose per kilogram per surgical minute was similar between the two groups (music group 0.05, control group 0.06; p= 0.264). Patient satisfaction scores with their perioperative experience were higher in the music group (p= 0.009).

Conclusions

Music therapy may be offered as an alternative to traditional anxiolytics intra-operatively. However further studies are warranted to evaluate whether or not music therapy can decrease sedation and anxiolytic medications during surgery. The type and mode of delivery of music also need to be studied to better understand the impact of music therapy.

Clinical trial registry: Clinicaltrials.gov # NCT03569397

## Introduction

Spinal anesthesia is being used more frequently for patients having knee arthroplasty because it is associated with better pain control, more stable vital signs, faster postoperative recovery, and fewer risks than general anesthesia [1]. However, the operating room environment, especially for orthopedic procedures, and more specifically for joint replacement surgery, can be loud since hammers and drills are often used. The sound of an orthopedic oscillating saw can reach up to 105 decibels (db) which may cause some degree of noise-induced hearing loss [2].

With spinal anesthesia, patients are more aware of this noise, potentially adding to the anxiety and necessitating the administration of additional sedating medications [3]. Higher sedation requirements during hip fracture repair are associated with a higher incidence of delirium in the elderly population [4]. Several studies have demonstrated that the use of headphones with patient-selected music can decrease anxiety in the operating room and improve overall patient satisfaction [5]. Listening to music during surgery has resulted in increased patient satisfaction and decreased sedation requirements for patients having other types of surgeries; however, this has not been studied in patients having total knee arthroplasty [6-7]. Music therapy is considered as a non-pharmacological intervention that can be used as an adjunct to decrease preoperative anxiety and also aid in postoperative recovery [8-9].

Perioperative anxiety can lead to increased pharmacological interventions, canceled procedures, and dissatisfaction [10]. The purpose of this study was to examine the effects of music therapy during spinal anesthesia for patients having total knee arthroplasty, specifically assessing if intraoperative music therapy impacts patient anxiety, sedation requirements during surgery, and postoperative satisfaction.

## Materials and methods

**Figure 1:**
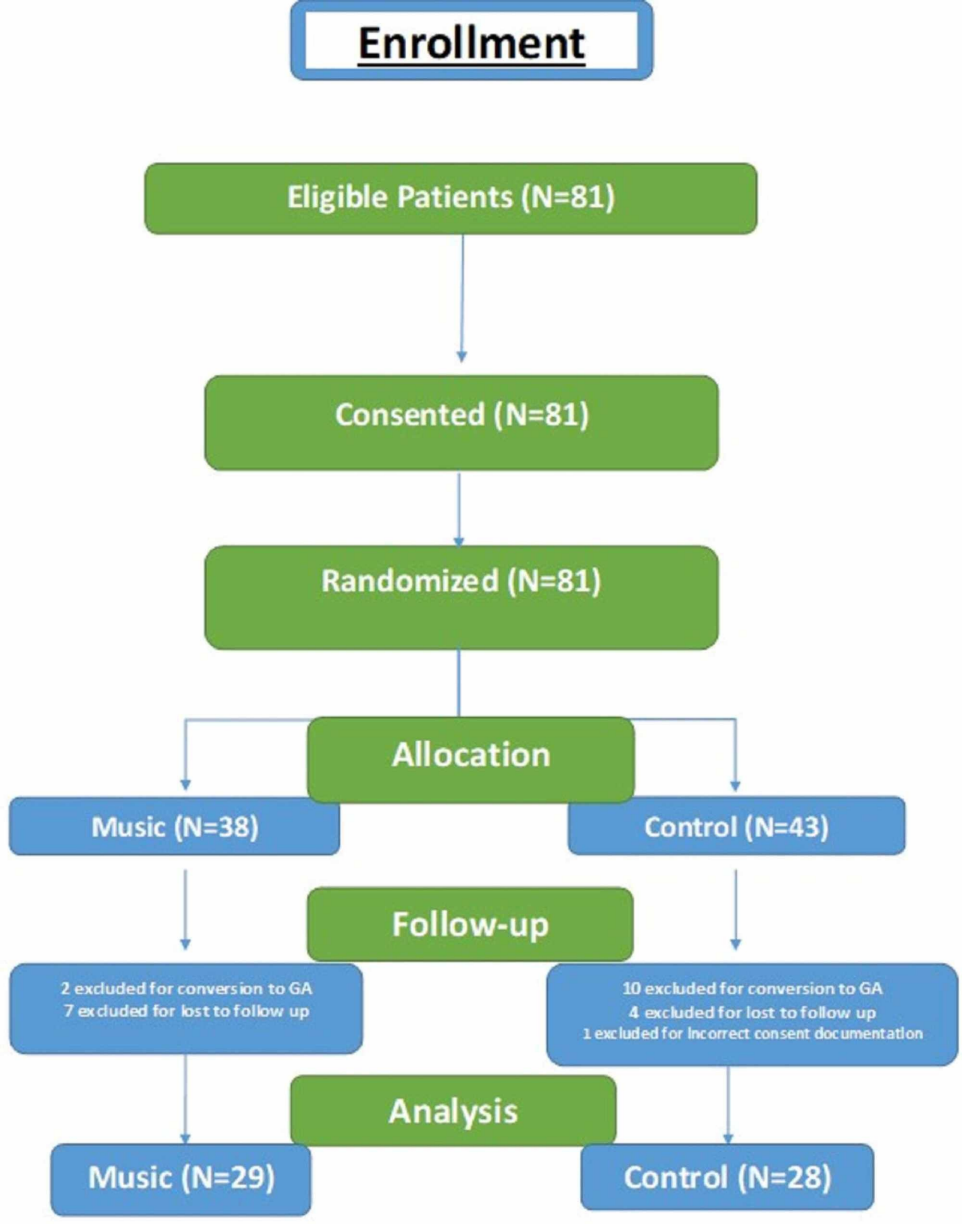
Consolidated Standards of Reporting Trials (CONSORT) flowchart of participant involvement

The prospective, randomized study received full institutional review board (IRB) approval and is registered at clinicaltrials.gov (NCT03569397). Patients greater than 18 years of age and of American Society of Anesthesiologists (ASA) Class I, II, or III undergoing total knee arthroplasty under spinal anesthesia were approached for participation. Patients with hearing impairment secondary to age or disease and any contraindications for spinal anesthesia were excluded from enrollment. Based on the criteria below, 81 patients were enrolled for the study (Figure 1). Upon informed consent, participants were randomized using computer-generated random numbers to either the investigational group (“Music” group) or the control group (“No Music” group). Anxiety scores were determined by the traditional validated tool, the State-Trait Anxiety Inventory (STAI) [11]. All patients completed a baseline STAI assessment preoperatively, which has a state and trait portion [12]. The patients in the music group chose a genre of music (blues, classical, country, gospel, jazz, classical, rhythm and blues, rock, soundtrack) and self-selected a volume level. Preoperatively, all patients in both groups received continuous adductor canal block for postoperative analgesia. All patients received 1 mg of midazolam and 50 mcg of fentanyl as sedatives for performing the nerve block.

Music was delivered through a pair of study-provided headphones. A Covidien Bispectral Index Monitor™ (BIS) (Medtronic Minimally Invasive Therapies, Minneapolis, MN) was used in both groups to confirm moderate sedation (65-75). A BIS reading of 70 is labeled as a general representation of moderate sedation in which the patient “may respond to loud commands.” Intraoperatively, all patients were given a standardized dose of 1 mg midazolam and 50 mcg fentanyl, as well as started on a propofol infusion. All patients received standard postoperative care, and on postoperative day 1, they again completed the STAI assessment, as well as a satisfaction survey. Descriptive analyses were completed on the outcome measurements of STAI scores, propofol dose, and reported patient satisfaction. Two sample t-tests were used to compare outcomes between study groups.

Sample size calculation and statistical analysis

The sample size for the primary outcome was calculated using a mean (standard deviation (SD)) STAI anxiety score of 33 and power of 80% to detect a clinically significant reduction in anxiety levels (4 points on anxiety tool) with a significance level alpha of 0.05. Continuous data were summarized as mean and standard error (SE) and categorical data were summarized as frequency and percentages. The assumption of normality for continuous variables were assessed using normal probability plots. The Statistical Analysis System (SAS), V.9.4 (SAS Institute, Inc., Cary, NC) was used to conduct all analyses. Two sample t-tests (for continuous variables) and Chi-square tests (for categorical variables) were used and a p-value of < 0.05 was considered statistically significant. Baseline characteristics were compared between the two groups using standard descriptive statistics.

## Results

**Figure 2:**
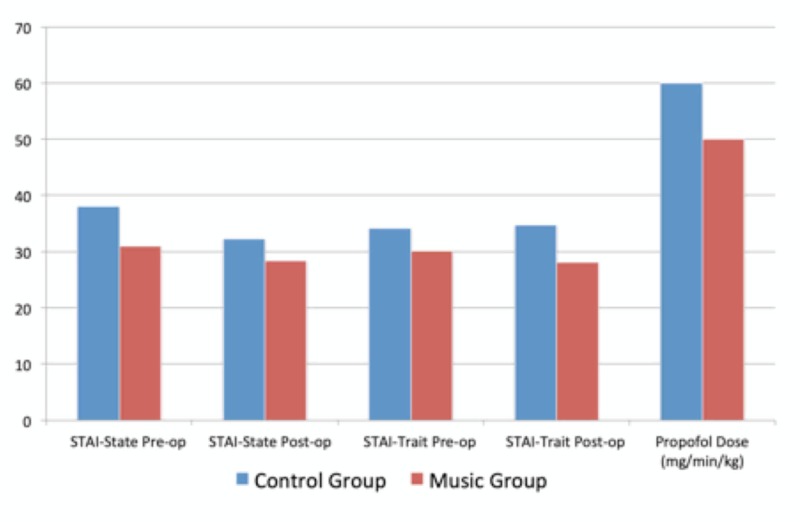
State-Trait Anxiety Inventory (STAI) scores and propofol dose in music and control groups

Fifty-seven participants completed the study (29 in the music group, 28 in the control group). Demographics can be found in Table 1. Statistical analysis of these values indicates that the groups were similar. Subjects in the music group had a statistically significant lower average STAI-State baseline score as compared to the control group (music group: 31.00 (SD 1.44); control group: 38.04 (SD 2.35); p = 0.01). Although not statistically significant, postoperative STAI-State scores for the music group were lower for the music group than the control group (music group 28.34 (SD 1.64); control group 32.21 (SD 1.56), p = 0.09) (Figure 2). STAI-Trait scores were similar preoperatively, but significantly less postoperatively in the music group (28.14, SD 1.0), as compared to the control group (34.71, SD 2.31); p = 0.01.

**Table 1:** Demographics and Case Information P refers to p-value with a statistical significance of p < 0.05. Gender and surgical side are calculated as percentages. Age, body mass index (BMI), and length of surgery are calculated as mean (standard deviation).

Characteristics	Control (n = 28)	Music (n = 29)	P
Gender			0.708
Female	18 (64.29%)	20 (68.97%)	
Male	10 (35.71%)	9 (31.03%)	
Age (years)	61.68 (2.27)	65.14 (1.82)	0.238
BMI (kg/m2)	33.14 (1.29)	34.80 (1.31)	0.369
Surgical side			0.881
Left	16 (57.14%)	16 (55.17%)	
Right	12 (42.86%)	13 (44.83%)	
Length of surgery (minutes)	97.11 (5.10)	98.62 (4.15)	0.818

When controlled for weight and duration of surgery, the propofol dose per kilogram per surgical minute was similar between the two groups (music group 0.05, control group 0.06; p = 0.264) (Table 2). Patient satisfaction scores with their perioperative experience were higher in the music group (p = 0.009), and when asked if listening to music helped to lessen anxiety and control pain, participants responded favorably. They also indicated that they would request to listen to music in the future and recommend it to their family and friends (Table 3). When asked, a majority of those in the control group wished that they had been given music (69%).

**Table 2:** Pre and Postoperative State-Trait Anxiety Inventory (STAI) and Propofol Doses P refers to p-value with a statistical significance of p < 0.05. SD: standard deviation

Surgical Characteristics	Control (n = 28)	Music (n = 29)	P
STAI State	Mean (SD)	Mean (SD)	
Preop	38.04 (2.35)	31.00 (1.44)	0.013
Postop	32.21 (1.56)	28.34 (1.64)	0.094
Change	5.82 (2.39)	2.66 (1.96)	0.309
STAI Trait			
Preop	34.21 (2.03)	30.10 (1.40)	0.100
Postop	34.71 (2.31)	28.14 (1.00)	0.011
Change	-0.50 (2.49)	1.97 (1.52)	0.398
Propofol dose per surgical minute per kg (mg/min/kg)	0.06 (0.004)	0.05 (0.004)	0.264

**Table 3:** Patient Satisfaction Survey Survey responses (music group only, n = 29) reported on a 5-point Likert scale from 1 (not at all) to 5 (extremely) SD: standard deviation

Question	Mean (SD)
Lessened anxiety	4.62 (0.12)
Provided calmness	4.88 (0.06)
Controlled pain	3.69 (0.29)
Personal attention	4.23 (0.21)
Request music in the future	4.73 (0.19)
Recommend to friends/family	4.77 (0.14)

## Discussion

Operative room (OR) noise-induced anxiety may be managed by administering larger doses of sedatives. These drugs can depress circulation and respiration, making non-pharmacologic alternatives attractive [13]. Music medicine as a non-pharmacologic intervention has demonstrated benefits in awake surgical procedures concerning anxiety and blood pressure [14]. Patients under general anesthesia also benefited from improved pain scores and patient satisfaction [15]. Music therapy can be a cost-effective and low-risk intervention to decrease the stress response to surgery and improve patient experience and satisfaction during total knee arthroplasty [16]. The noise levels in the OR are alarmingly high and are routinely higher than the limits established by institutional or federal regulations [17]. This randomized, controlled study has confirmed the above findings of higher patient satisfaction and subjective lowering of anxiety levels. A unique finding in this study was the difference in the STAI-State scores between the two groups indicating a random group that started the study with less anxiety than the control group. While both groups did show a decrease in the STAI-State score, a larger benefit may be obscured by a preoperative score of 31 that starts below previously reported averages between 33 and 35 [18]. Including preoperative anxiety measurements as a variable may be necessary to clarify those patients who would most benefit from music therapy. Further investigation with a larger sample size is warranted to determine if sedation requirements may be reduced. Additionally, a more longitudinal use of music, such as in the preoperative or postoperative setting, could be considered.

Compared to the control group, music therapy was not found to reduce propofol requirements according to BIS monitor target range for moderate sedation. This result may reflect the ineffectiveness of the intervention on this particular variable, or it may be due to the limitations of the BIS monitoring. Although the BIS monitor is a preferred tool to assess the sedation level [19], clinical scientists may investigate alternate methods to evaluate the effect on music on sedation requirements.

Another interesting observation from this study (CONSORT flowchart) revealed that 23% of allocated “control” group patients were converted to general anesthesia as compared to only 5% in the “music” group. This could be explained by plausible lower anxiety and pain levels in the “music” group intra-operatively. We plan to study this finding in a separate retrospective study to confirm and understand it better.

Further objective research is necessary to elucidate the evidence regarding the therapeutic value of music therapy [20]. This information may provide a basis for research in perioperative settings to evaluate the use of music therapy in the management of anxiety in various surgical populations.

## Conclusions

Music therapy in this study did not decrease propofol requirements for sedation during total knee arthroplasty. A larger sample size is warranted to validate this finding and the effect of music therapy on anxiety levels post-intervention. Also, further studies to study the effect of music therapy on the conversion of spinal to general anesthesia for total knee arthroplasty are warranted.

## References

[1] Bansal P, Kharod U, Patel P, Sanwatsarkar S, Patel H, Kamat H (2010). The effect of music therapy on sedative requirements and haemodynamic parameters in patients under spinal anaesthesia; a prospective study. J Clin Diagn Res.

[2] Simpson JP, Hamer AJ (2017). How noisy are total knee and hip replacements?. J Periop Pract.

[3] Koelsch S, Fuermetz J, Sack U (2011). Effects of music listening on cortisol levels and propofol consumption during spinal anesthesia. Front Psychol.

[4] Sieber FE, Zakriya KJ, Gottschalk A, Blute MR, Lee HB, Rosenberg PB, Mears SC (2010). Sedation depth during spinal anesthesia and the development of postoperative delirium in elderly patients undergoing hip fracture repair. Mayo Clin Proc.

[5] Ilkkaya NK, Ustun FE, Sener EB (2014). The effects of music, white noise, and ambient noise on sedation and anxiety in patients under spinal anesthesia during surgery. J Perianesth Nurs.

[6] Koch ME, Kain ZN, Ayoub C, Rosenbaum SH (1998). The sedative and analgesic sparing effect of music. Anesthesiology.

[7] Lepage C, Drolet P, Girard M, Grenier Y, DeGagné R (2001). Music decreases sedative requirements during spinal anesthesia. Anesth Analg.

[8] Bringman H, Giesecke K, Thörne A, Bringman S (2009). Relaxing music as pre-medication before surgery: a randomized controlled trial. Acta Anaesthesiol Scand.

[9] Hole J, Hirsch M, Ball E, Meads C (2015). Music as an aid for postoperative recovery in adults: a systematic review and meta-analysis. Lancet.

[10] Thomas T, Robinson C, Champion D, McKell M, Pell M (1998). Prediction and assessment of the severity of postoperative pain and of satisfaction with management. Pain.

[11] Spielberger CD (2010). State-Trait Anxiety Inventory for adults. The Corsini Encyclopedia of Psychology.

[12] Julian LJ (2011). Measures of anxiety: State-Trait Anxiety Inventory (STAI), Beck Anxiety Inventory (BAI), and Hospital Anxiety and Depression Scale-Anxiety (HADS-A). Arthritis Care Res (Hoboken).

[13] Walther-Larsen S, Diemar V, Valentin N (1988). Music during regional anesthesia: a reduced need of sedatives. Reg Anesth Pain Med.

[14] Wu PY, Huang ML, Lee WP, Wang C, Shih WM (2017). Effects of music listening on anxiety and physiological responses in patients undergoing awake craniotomy. Complement Ther Med.

[15] Kahloul M, Mhamdi S, Nakhli MS, Sfeyhi AN, Azzaza M, Chaouch A, Naija W (2017). Effects of music therapy under general anesthesia in patients undergoing abdominal surgery. Libyan J Med.

[16] Gooding L, Swezey S, Zwischenberger JB (2012). Using music interventions in perioperative care. South Med J.

[17] Katz JD (2014). Noise in the operating room. Anesthesiology.

[18] Graff V, Cai L, Badiola I, Elkassabany NM (2019). Music versus midazolam during preoperative nerve block placements: a prospective randomized controlled study. Reg Anesth Pain Med.

[19] Newman A, Boyd C, Meyers D, Bonanno L (2010). Implementation of music as an anesthetic adjunct during monitored anesthesia care. J Perianesth Nurs.

[20] Marwick C (2000). Music therapists chime in with data on medical results. JAMA.

